# Blockchain for Increased Trust in Virtual Health Care: Proof-of-Concept Study

**DOI:** 10.2196/28496

**Published:** 2021-07-30

**Authors:** Anton Hasselgren, Jens-Andreas Hanssen Rensaa, Katina Kralevska, Danilo Gligoroski, Arild Faxvaag

**Affiliations:** 1 Department of Neuromedicine and Movement Science Norwegian University of Science and Technology Trondheim Norway; 2 Department of Information Security and Communication Technology Norwegian University of Science and Technology Trondheim Norway

**Keywords:** blockchain, ethereum, decentralization, Healthcare 4.0, virtualization, trust

## Abstract

**Background:**

Health care systems are currently undergoing a digital transformation that has been primarily triggered by emerging technologies, such as artificial intelligence, the Internet of Things, 5G, blockchain, and the digital representation of patients using (mobile) sensor devices. One of the results of this transformation is the gradual virtualization of care. Irrespective of the care environment, trust between caregivers and patients is essential for achieving favorable health outcomes. Given the many breaches of information security and patient safety, today’s health information system portfolios do not suffice as infrastructure for establishing and maintaining trust in virtual care environments.

**Objective:**

This study aims to establish a theoretical foundation for a complex health care system intervention that aims to exploit a cryptographically secured infrastructure for establishing and maintaining trust in virtualized care environments and, based on this theoretical foundation, present a proof of concept that fulfills the necessary requirements.

**Methods:**

This work applies the following framework for the design and evaluation of complex intervention research within health care: a review of the literature and expert consultation for technology forecasting. A proof of concept was developed by following the principles of design science and requirements engineering.

**Results:**

This study determined and defined the crucial functional and nonfunctional requirements and principles for enhancing trust between caregivers and patients within a virtualized health care environment. The cornerstone of our architecture is an approach that uses blockchain technology. The proposed decentralized system offers an innovative governance structure for a novel trust model. The presented theoretical design principles are supported by a concrete implementation of an Ethereum-based platform called VerifyMed.

**Conclusions:**

A service for enhancing trust in a virtualized health care environment that is built on a public blockchain has a high fit for purpose in Healthcare 4.0.

## Introduction

### Overview

As a result of health care development, societies are undergoing a current demographic shift—people live longer, and fewer are born. The overall increase in life expectancy between 1970 and 2013 was 10.4 years on average for Organization for Economic Cooperation and Development countries [1]. A direct effect of this demographic shift [2,3] is that noncommunicable and chronic diseases become more prevalent, which presents a substantial socioeconomic challenge. Consequently, fewer caregivers need to support an ever–increasing number of retirees with a rising number of chronic diseases. This unsustainable scenario is the strongest motivation behind many different ongoing proposals for transformations in the health care industry. Delivering health care, as we know it today, will most likely be unaffordable for any health system in 15 years from now, and many health services will have to be delivered by nonprofessionals and machines. This includes artificial intelligence health workers and devices connected via machine-to-machine protocols and automated, computerized services, which will be accessible via fast connections from anywhere, anyhow, and at any time (5G).

Furthermore, individuals will be forced to take more responsibility for their own health, take preventative measures, seek proper care in a timely manner, and behave more like autonomous patients. To facilitate this, there is a need to provide the right tools to encourage and enforce this transformation, both from the delivery side (health care providers) and the receiver side (patients). This transformation toward Healthcare 4.0 will challenge many of the present key components in a functional health system, where the concept of trust is one.

The first contribution of this paper is to review and predict the evolution of health care, and to identify the potential problems that could emerge in this transformation. It forms a theoretical foundation and urges the need for novel solutions to enhance trust. Second, the presented theoretical design principles are supported by the concrete implementation of a proof of concept. For this contribution, we choose the cornerstone in our architecture to be a blockchain technology implemented as an Ethereum-based platform called VerifyMed.

The remainder of this paper is organized as follows: the first section introduces blockchain and previous related work; the next section presents the method applied in this work; the following section outlines the results of technology forecasting and presents a trust issue in a virtualized health care environment; *the next* section presents a novel blockchain-based trust model for competence verification of health care personnel, and the final section provides a discussion and conclusions of the work.

### Related Work and Blockchain Overview

**Figure 1:**
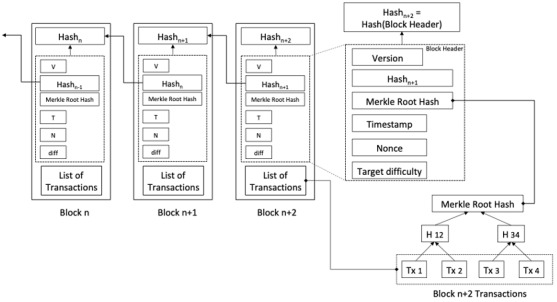
A generic overview of a blockchain structure.

Blockchain can be seen as an unconventional platform that alleviates the reliance on a single, centralized authority, yet it still supports secure and pseudoanonymous (or anonymous) transactions and agreements directly between interacting parties. It offers various degrees of decentralization, immutability, and consensus firmly founded in the mathematical principles of modern cryptography. A blockchain can also be described as an immutable ledger that logs data entries in a decentralized manner. In its original form, a blockchain enables entities to interact without a central trusted third party. The blockchain consists of a continuously growing set of data entries bundled together into blocks of data (Figure 1). Upon acceptance of the blockchain, these blocks are linked to the previous and future blocks sequentially [4]. In blockchain’s original definition, this ledger of data blocks is decentralized and distributed across many nodes. This distributed ledger is transparent, verifiable by all, and tamper-proof. Owing to these properties, the blockchain has gained much attention for various applications. The first use case of a blockchain, Bitcoin, was introduced by a person or a group under the name of Satoshi Nakamoto in 2008 [5]. Bitcoin is also known as *a cryptocurrency.* Although cryptocurrencies remain the primary use case for blockchain, there is a substantial interest in applying this technology for other purposes and sectors [6]. Additionally, a blockchain allows for smart contracts—self-execution contracts that do not require any central authority. The use cases of blockchain in the health domain are increasing exponentially, as shown by Hasselgren et al [7], among others.

Blockchain technology has five fundamental attributes that define the technology: (1) distribution, (2) decentralization, (3) time stamping, (4) data provenance, and (5) nonrepudiation. These five attributes are applied when addressing the fundamental problems in health care informatics and are part of driving the transformation toward Healthcare 4.0. The first generation of blockchain platforms led by Bitcoin [5] had a specially defined programming language for users to construct different transactions in the blockchain. The initial design rationale was that the programming language should be as simple as possible to satisfy the needs for building various transaction types and should not be a fully developed and powerful programming language. In computer science, the category of powerful programming languages is called the *Turing Complete*. The first blockchain platform that offered a Turing Complete language for programming, not just simple transactions but also more complex *smart contracts* and fully developed apps, was Ethereum [8]. There is an active debate on which concept is better and safer—development of malicious programs for blockchain platforms that do not have the Turing Complete programming language is very difficult and limited, in contrast to blockchain platforms that have the Turing Complete languages [5,9]. Nevertheless, it seems that the blockchain platforms that come with a fully developed Turing Complete programming language are very suitable for developing decentralized applications (dApps) for Healthcare 4.0, which is further elaborated in the next section.

### Blockchain Platforms, dApps, and Smart Contracts

There are several decentralized platforms and frameworks for building dApps. Ethereum is the most common in health care applications [10]. This is most likely due to the large number of developers in the Ethereum community. Nevertheless, Ethereum has proven to be a solid platform for health care dApps [11]. Compared with the first and largest blockchain to date—Bitcoin—Ethereum has incorporated smart contracts, a function that substantially opens up the features of dApps built on Ethereum.

Smart contracts can be considered as self-executing contractual agreements, where preagreed upon provisions are formalized in the source code. Smart contracts can be automatically enforced based on these preagreed provisions, and they can work without any third party. The functions within a smart contract can be awoken in a blockchain transaction, and the use of this functionality could appeal to the health domain [8].

Zhang et al [11] stated that a well-designed health care dApp should limit the storage of encrypted sensitive data on the blockchain. Furthermore, they recommend that a dApp dealing with health care data should support Turing completeness to facilitate communication among various parties and handle the exchange of sensitive patient data. In the study by Kuo et al [10], there were clear indications that Ethereum, Hyperledger, and Multichain are more suitable platforms for the health domain than other blockchains.

Blockcerts [12] is a standard developed for verifying certificates of competence by storing signatures on a blockchain. The standard relies on existing trust relationships between the issuer and verifier of the certificate. Baldi et al [13] showed that certificates within this system could be spoofed. They also proposed the use of decentralized identifiers to govern such certificates. At present, there are private initiatives for medical credentials that use a blockchain. The first on the market was ProCredEx by Hashed Health [14]. They state that they have developed a blockchain-based solution that enables faster onboarding and credential verification.

Furthermore, a newly introduced collaborative project between Axuall, Inc and Metro-Health [15] announced a service for credentials of clinical practitioners. They state that they will enable digital portfolios that will include documentation of a practitioner’s education, specialty training and board certifications, licenses, sanctions or medical malpractice judgments, evaluations, work history, and hospital affiliations. As these are private endorsers, there is no published peer-reviewed literature on their technical solutions. In addition to what is mentioned above, based on our knowledge, there is no published research that has addressed the same scope as our framework. As described earlier, several private organizations have created solutions for medical credentials by using blockchain technology. However, these are all based on the United States and are somewhat tailored to the US health system. We explore a broader solution in the form of a decentralized trust model that addresses the current issues with board certificates and credentials and creates an immutable portfolio of completed clinical work by a health care professional that is verifiable by all. The research approach used in this study is described in the following section.

## Methods

### Overview

**Figure 2:**
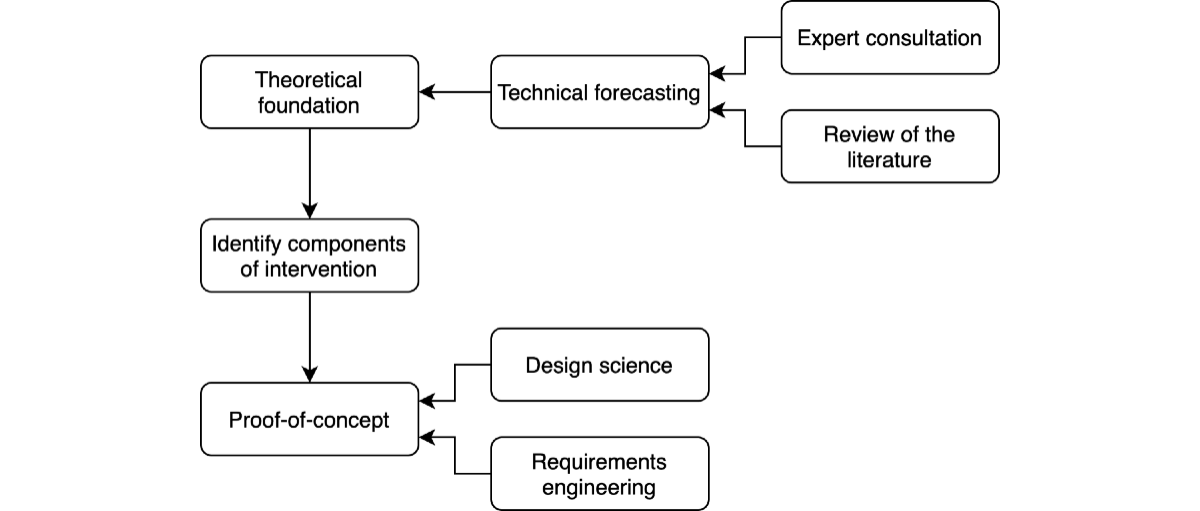
Research approach for the presented work.

The research approach used in this study, as shown in Figure 2, follows one of the frameworks presented by Campbell et al [16], which describes a framework for the design and evaluation of complex intervention research within health care. Our study aims to address the following two key issues outlined in the framework: (1) establish the theoretical basis of the intervention and (2) identify and describe the components of the complex intervention.

### Summary of Knowledge and Technology Forecasting

We applied the most common method for addressing technology forecasting by reviewing the current literature and consulting domain experts [17]. The domain expert consultation was conducted in an unstructured manner; a convenient sample of (health) informatics experts from Norway was consulted about their views on the future of health care and Healthcare 4.0. A scoping review of the literature on the future of health care was performed in a semisystematic manner, and this is described in the section *Summary of Knowledge: Healthcare 4.0*.

### Identifying Components of the Intervention

On the basis of the forecasting of Healthcare 4.0, a potential trust challenge is described and elaborated as the primary component of the intervention. This is presented in section *Trust in Healthcare 4.0*.

### Proof of Concept

Furthermore, our work presents some technical components of a proof of concept to conceptualize (1) and (2), following the principles of design science [18] and requirements engineering [19]. This is presented in the section *VerifyMed: A Novel Trust Model*.

## Results

### Overview

We first describe a technology forecast of health care and then demonstrate how trust will emerge in this transformed health care system as a component of an intervention. The proof-of-concept VerifyMed is presented in a separate section, that is, *VerifyMed: A Novel Trust Model*.

### Summary of Knowledge: Healthcare 4.0

Healthcare 4.0 [20] is a strategic concept for the health domain derived from the Industry 4.0 concept. The aim of Healthcare 4.0 is to allow for advanced virtualization to enable the personalization of health care in real time for patients, professionals, informal health workers, and nonhuman health workers. A transformation toward Healthcare 4.0 will be a shift from hospital or professional-centered health care (patient in hospitals) to a globalized, virtualized, and self-administered health care via distributed patient-centric care (multiple care providers) and later to patient-driven care fueled by personally generated health data.

Lasi et al [21] define Industry 4.0 with a wide range of current concepts: smart factories, cyber-physical systems, self-organization, new systems in distribution and procurement, new systems in the development of products and services, adaptation to human needs, and corporate social responsibility. Similarly, this categorization has been applied to health system development in the Healthcare 4.0 concept.

Thuemmler and Bai [20] state that:

The aim of Healthcare 4.0 is to allow for progressive virtualization in order to enable the personalization of healthcare next to real-time for patients, professionals, and formal and informal caregivers. The personalizing of healthcare will be achieved through the massive use of cyber-physical systems, cloud/edge computing, the Internet of everything including things, services and people and evolving mobile communication networks (5G).

The six design principles from Industry 4.0 could be applied to Healthcare 4.0 to forecast health care transformation and to design applications with a high fit for purpose. The following design principles were proposed [22]:

1. Interoperability: enable people and machines to communicate through data standards and standardized infrastructure.
2. Virtualization: technologies for interoperability, faster internet connections, and connected devices enable the movement of parts of the physical processes in health care to a virtual environment.
3. Decentralization: linking real-time data and users together opens up more autonomous decisions and reduces the necessity of centralized services.
4. Real-time capability: a higher proportion of connected devices and people enables changes in real time.
5. Service orientation: a shift from products to services based on accumulating data could adapt faster to market changes.
6. Modularity: a higher degree of module-based delivery and configuration enables faster adoption of changing needs.

From an academic perspective, design principles are the foundation of the design theory [23]. As outlined in the section *Proof of Concept*, the design theory method is followed in developing our proof-of-concept platform, VerifyMed.

The following section presents an emerging problem in Healthcare 4.0, which serves as the basis for the components of our intervention.

### Trust in Healthcare 4.0

#### Overview

The definition of trust is a broad, multilayered, and complex concept that varies depending on the academic discipline that uses the term [24]. For this study, we have adopted the following broad definition of trust: *a psychological state comprising the intention to accept vulnerability based upon positive expectations of the intentions or behavior of another* [25].

#### Trust From a Human Psychology Perspective

A central part of clinical practice is trust between a patient and a health care professional [26]. Maintaining trust with patients is a core function for physicians in their clinical practice [27]. The General Medical Council states “patients must be able to trust doctors with their lives and health” [28]. This is also a part of the obligations of other health care professionals such as nurses [29]. Trust in health care professionals is considered a foundation for effective service delivery [30] and a core attribute in patient-centered care [31].

Commonly, trust is divided into interpersonal, social, and dispositional trust [32].

Furthermore, trust between a trustor and a trustee is encouraged by the trustee’s reliability (good reputation), competence (having skills to perform the task at hand), and integrity (honesty) [33]. Trust in a physician is related to increased treatment adherence, patient satisfaction, and improved health status [34]. Patients most commonly base their trust on doctor’s characteristics such as competence, compassion, privacy and confidentiality, reliability and dependability, and communication skills [35].

**Figure 3:**
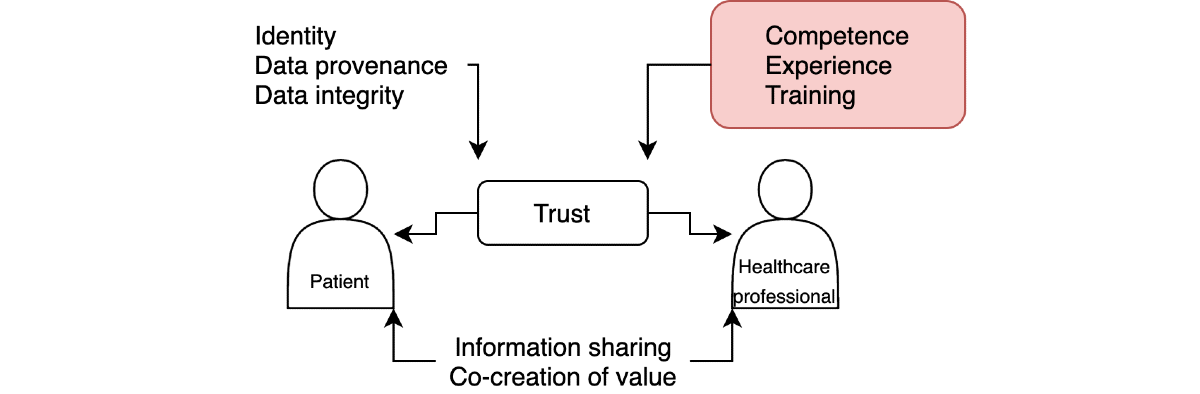
Factors influencing trust in a patient-caregiver interaction.

We know from other industries that a successful web-based consultation in health care delivery service requires a value cocreation between the caregiver and the patient [36]. Caregivers need active participation from patients to benefit from this cocreation. Several factors contribute to the trust foundation, which is the basis for value creation, as illustrated in Figure 3. Our approach targets the verification of competence, experience, and training (highlighted in Figure 3).

#### Trust in Medical Technologies

When trusting medical technologies, institutional trust and technical reliability are deeply intertwined [37]. A key takeaway when reviewing Industry 4.0 is the need to explore further and understand how to build trust in the context of digital and virtualized health. This is related to trust in systems and information (human system) and people having the control of sharing information (human-human through the system).

#### Trust Issues in Healthcare 4.0

To conceptualize one part of the trust ecosystem in health care, we present the following theoretical issues with trust in a virtual patient-caregiver relationship: the patient needs to trust that the caregiver has the right competence (and authority) to deal with his or her health problem in a physical as well as in a virtualized health care environment. The caregiver needs to show the patient that he or she possesses the right competence to deal with the health problem of that specific patient; otherwise, the patient will possibly go somewhere else.

There are currently few or no systematic and objective tools to verify the competence and experience of health professionals in a transparent and accessible manner. The records of cases of delivered care are often stored in the electronic health record of the respective hospital. If a health care worker changes an employer, there is little or no opportunity to bring the ledger of given care (work experience). Like other industries, the health care industry has experienced a fast turnover of personnel. More health care workers change employers at a faster rate [38]. More health care workers are also moving across borders and jurisdictions at an increasingly higher pace [39]. In these cases, a tamper-proof, accessible record of the work history of someone as a health care professional, owned and controlled by no single entity, could be valuable. If this *portfolio* was stored in a decentralized manner, easily accessible with the consent of the particular health care worker, onboarding processes for employers in the health care domain could be improved and that the health care worker could feel confident in that they control their own reputation by providing evidence-based care that could be verified at any time.

There is a need for patients, health care workers, and health care facilities to be able to verify the skill, competence, and formal certificates of health care personnel, especially when health care is moving toward Healthcare 4.0. Furthermore, it is essential to create an audit trail of complete work for health care workers; this could function as a portfolio that could potentially be used for future employers, freelance work, and increased confidence among health care workers.

**Figure 4:**
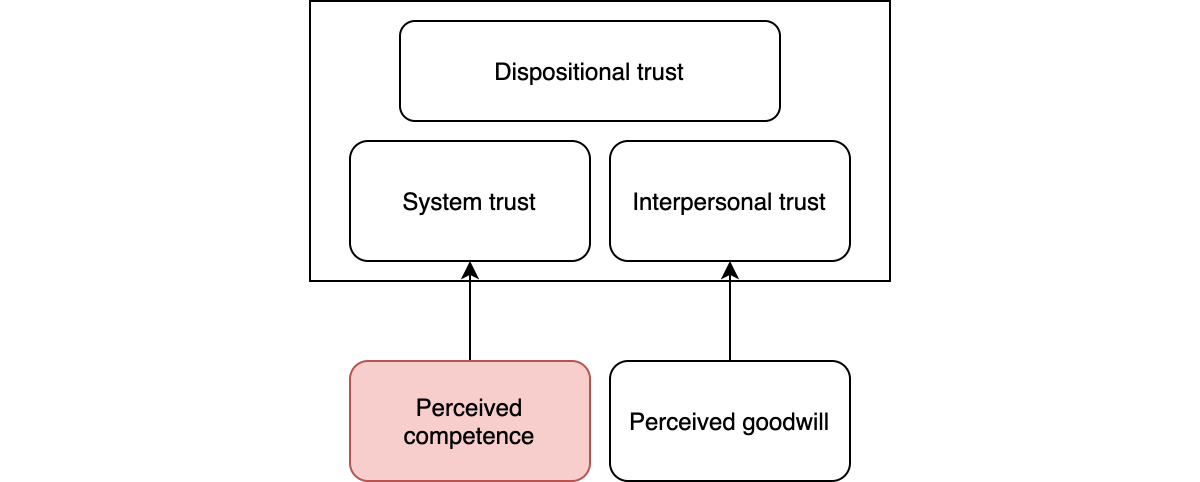
Trust model (adapted from Leimeister et al [32]).

Previous work has concluded that perceived competence and perceived goodwill are contributing factors to the system and interpersonal trust [24,32]. In a virtualized health care environment, it becomes increasingly important to verify the competence and credentials of health care professionals, as perceived competence is an essential component in building trust [40,41]. This highlighted component of perceived competence in Figure 4 is one part that the concept of VerifyMed partially addresses.

The following section presents a proof of concept that addresses those needs.

### VerifyMed: A Novel Trust Model

#### Overview

Our proposed architecture’s technical core and the operational functionality are described in the studies by Rensaa et al [42-44]. In addition to that technical part, we describe some of the crucial functional and nonfunctional requirements and the principles that influenced our design rationale.

Our proposed architecture provides a solution for enhancing trust between a caregiver and a patient within a virtualized health care environment. The cornerstone feature in our architecture is its ability to capture trust relationships within the health care system and put them in a blockchain. Patients can use this trust mechanism to confirm credentials and potentially enhance trust in a caregiver during their interaction. Furthermore, the architecture includes tools for evaluating these interactions publicly on a blockchain. These evaluations served as a file for the caregiver’s experience. We proposed the following three types of evidence for building trust in a virtualized health care environment: evidence of authority, evidence of experience, and evidence of competence [43].

The functional requirements describe the key features that we desire in our system based on our problem statement. Nonfunctional requirements describe the properties of the system, such as security, privacy, and performance requirements. Nonfunctional requirements often have a sizable architectural impact on how the system is implemented, whereas the functional requirements present the functionality that should be present within the architecture. These requirements are deduced from both industry requirements for handling patient data and the perceived problems deduced from our problem statement.

Previous research on blockchain apps within the health care industry has defined general principles for the requirements and system design principles that should be followed. Zhang et al [11] defined the metrics for evaluating blockchain apps within the health care industry. Although they are primarily directed toward the American Health Insurance Portability and Accountability Act (HIPAA), we generalize and try to capture some of these principles in our requirements.

#### Regulatory Compliance: Compliance With Current Health Data Laws and (Health) Privacy Regulations

Several regulatory bodies are responsible for preserving privacy and access rights to personal health data. The most prominent are the HIPAA for the United States and the General Data Protection Regulation (GDPR) for the European Union. In addition, most countries have national health data laws that further regulate health data for their citizens. In the scope of this study, we explored the GDPR compliance for VerifyMed. There are currently some uncertainties around general blockchain compliance with the GDPR [44], and these uncertainties, mainly around the level of anonymization and identification of data controllers in a decentralized network, have not yet been clarified in any court case by the European Data Protection Board. However, it has been argued that there are no compliant blockchains, only complaint use cases, and apps [44]. The VerifyMed platform is designed to enhance user privacy and access control, and the following relevant GDPR articles have been addressed [45].

The VerifyMed platform is also designed to enhance the right of access by the data subject (Article 15 of the GDPR). As the system is designed not to store any personal data on the blockchain, it is also compliant with Article 17 of the GDPR (right to erasure or “right to be forgotten”), which only refers to personal data.

As the system is decentralized by design and there are possibilities for the user to access and receive the data at any time, it is compliant with Article 20 of the GDPR (right to data portability). The system requires an identity management solution to ensure full anonymization of the users and complies with Article 32 of the GDPR (security of processing). Identity management is not addressed within the scope of this study.

#### Key Functional Requirements

In accordance with the patient-centric health care system, we chose to define our main functional requirements in the context of the patient. As will be described later, the blockchain component of our architecture can be defined as a provider-centered model. We also note that fulfilling our patient-centered requirements allows the architecture to be used in settings outside of the patient and caregiver relationship. The main purpose of the model was to serve a patient-centered use case. The key patient-centered functional requirements were as follows:

1. Verification of caregiver credentials: a patient using a third-party system to talk with a caregiver should be able to verify the credentials by only using data from the blockchain. The patient must be able to do so without relying on any trust in the medical professional.
2. Verification of caregiver experience: a patient should be able to evaluate the experience of a medical professional by looking at data from the blockchain. Thus, the credibility of the data on blockchain must be enforced. The presented patient-centered requirements trigger opinionated system design choices to support this functionality. We additionally define two key features and refer to them as other deduced requirements. Therefore, these features will be subject to further specifications through nonfunctional requirements.
3. Transparency of blockchain data: to support data transparency to patients, we chose to use a publicly available blockchain to store the blockchain data. As these blockchains often have an associated fee with transactions, the system must take this into account.
4. Governance of blockchain data: to ensure that the trust relationships on the blockchain are anchored in the real world, they should be anchored in the existing corresponding trust relationships within the health care system. Just as there are governance entities responsible for credentials in the real world, they should be present in the proposed architecture as well.

#### Nonfunctional Requirements (via Quality Attributes)

##### Overview

In addition to the functional requirements above, we also surface the nonfunctional attributes of the system through quality attributes. The number of quality attributes of a system is unbounded. Therefore, this section presents the quality attributes that are considered to have the most significant architectural impact on the system.

##### Security Requirements

###### Fraudulent Treatments

A treatment cannot be published in the blockchain by unauthorized parties. All treatments must be cryptographically protected by an entity with direct or implicit authority to publish treatments.

###### Fraudulent Treatment Approvals

A treatment cannot be approved on the blockchain by unauthorized parties. All treatments must be approved by a license holder who the patient approves.

###### Fraudulent Evaluation

It should be impossible to publish an evaluation without going through a valid treatment. Once treatment has a related patient-reported outcome measure (PROM) published, it should not be possible to create another PROM related to the same treatment.

###### The Integrity of Treatments

It must be possible to ensure that a treatment or evaluation has not been tampered after their publication to ensure the credibility of these data sets. It is possible to prove this by using blockchain data.

##### Privacy Requirements

###### Unlinkability to Patients

The identity of patients must be treated as confidential. It should not be possible to link a transaction on the blockchain to a specific patient without any further knowledge from outside the blockchain. This will contribute to making the proposed system GDPR and HIPAA compliant (reference to regulatory compliance).

###### The Anonymity of Patients

The content of evaluations and treatments published on the blockchain should not reveal the identity of patients. The data published on the blockchain should either be a summary that cannot be linked to the patient or in another format that cannot be linked to a specific patient.

###### Access to Patient Data

The complete evaluations, including data linkable to patients, should be stored outside the blockchain. These data sets should be used to control patients. Access to these data sets for entities outside the patient and caregiver interaction should be denied unless the patient grants access.

##### Availability Requirements

###### Addition of New Governance Entities

It should be possible to add new governance entities dynamically without any code changes to the original contracts on the blockchain.

###### Recoverability After Authority Loss

If a governance entity becomes permanently unavailable or misbehaves, it should be possible to remove it, that is, to recover the dApp into a healthy state without interaction from the misbehaving authorizing entity.

##### Scalability Requirements

*The amount of data on the blockchain should be minimal*: the public blockchain is an expensive storage medium. Small data formats and encoding should be used to represent the data in the blockchain.

##### Performance Requirements

*Minimization of transactions*: interactivity with the blockchain should be reduced. The number of transactions required to go from the start to the published PROM should be small.

#### The Architecture

##### Overview

Our novel architecture provides trust between caregivers and patients within a virtualized health care environment. This is done through three main processes: evidence of authority, evidence of experience, and evidence of competence, each with its own components and stakeholders associated with them. We first define the terminology used in our architecture. Second, we present our proposition through an overall reference architecture. Finally, we describe how we further refine the reference architecture. We do this by describing the processes in the order in which they occur in the real world, along with the main components associated with them.

##### Terminology

**Figure 5:**
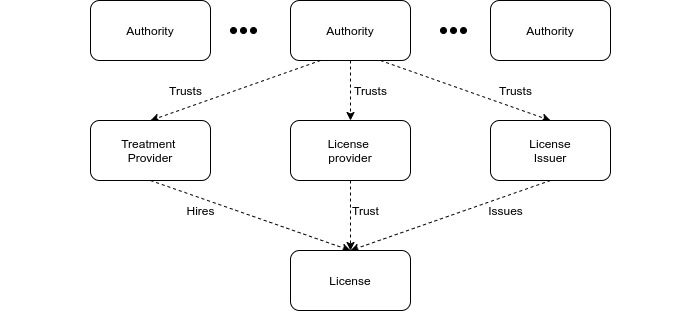
Trust relationships published on the blockchain.

Our architecture uses a concept for many different stakeholders, each represented by a given terminology. The stakeholders shown in Figure 5 are defined as follows:

1. Authorities: these are top-level government actors that have the overall responsibility of the health care sector (eg, national health directorates).
2. License: a license represents the practitioner’s role as health personnel. Although a license in a traditional sense is the authorization of health personnel, we instead use it to represent the personnel themselves. Authorization is captured through trust relationships related to licenses.
3. License issuer: organizations that issue licenses for health personnel. License issuers are the only ones that can create licenses.
4. License provider: organizations that give formal authorization to practice for a license.
5. Treatment provider: organizations in which practitioners operate and are responsible for issuing treatments for patients. Examples include hospitals, clinics, and virtualized health care services.

##### Overall Reference Architecture

###### Overview

**Figure 6:**
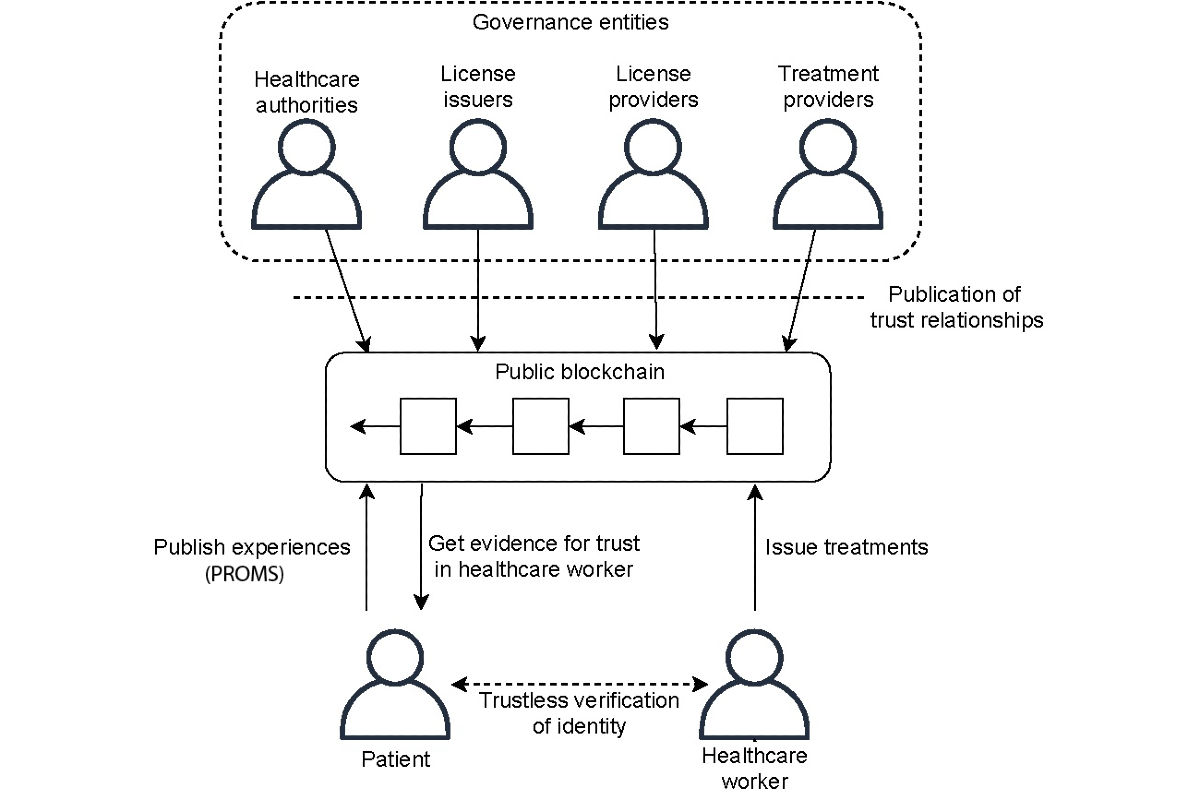
The VerifyMed architecture for providing trust in a virtualized health care environment. PROMS: patient-reported outcome measures.

As described in the functional requirements, the goal of VerifyMed is to provide trust in a health worker from a patient’s perspective. The high-level reference architecture is shown in Figure 6. It captures trust by using a blockchain to store the formal trust relationship from health care organizations to health workers. Furthermore, as health workers issue treatments over time, summaries of these are published on blockchain. Finally, the evaluations of these treatments were published on the blockchain. The result is that the formal credentials of a practitioner can be validated through trust relationships, and their experience can be captured through logged treatments and evaluations.

###### Creating Trust in a Caregiver

The first goal of the architecture is to capture the formal trust relationships between organizational actors and care providers within the health care industry. The end goal is to form a deduced trust relationship from health care authorities to the care provider and to capture the relationship in a way that is transparent and can be validated by the patients.

Figure 5 describes our model for trust relations between organizations and care providers. The top level was composed of large health care authorities. Authorities organize themselves through a model of distributed governance, for example, through simple voting, where existing trusted authorities can vote for the addition or removal of an authority. The main role of authorities is to provide trust in the defined stakeholders, who issue, authorize, and hire license holders. License holders can only practice and otherwise interact with the blockchain if all their upstream relations are linked to an authority. The patient entity is not part of this trust hierarchy; that is, patients are invited to publish PROMs on the blockchain by the care providers who have a trusted license after a completed treatment or interaction.

###### Caregiver and Patient Interaction

**Figure 7:**
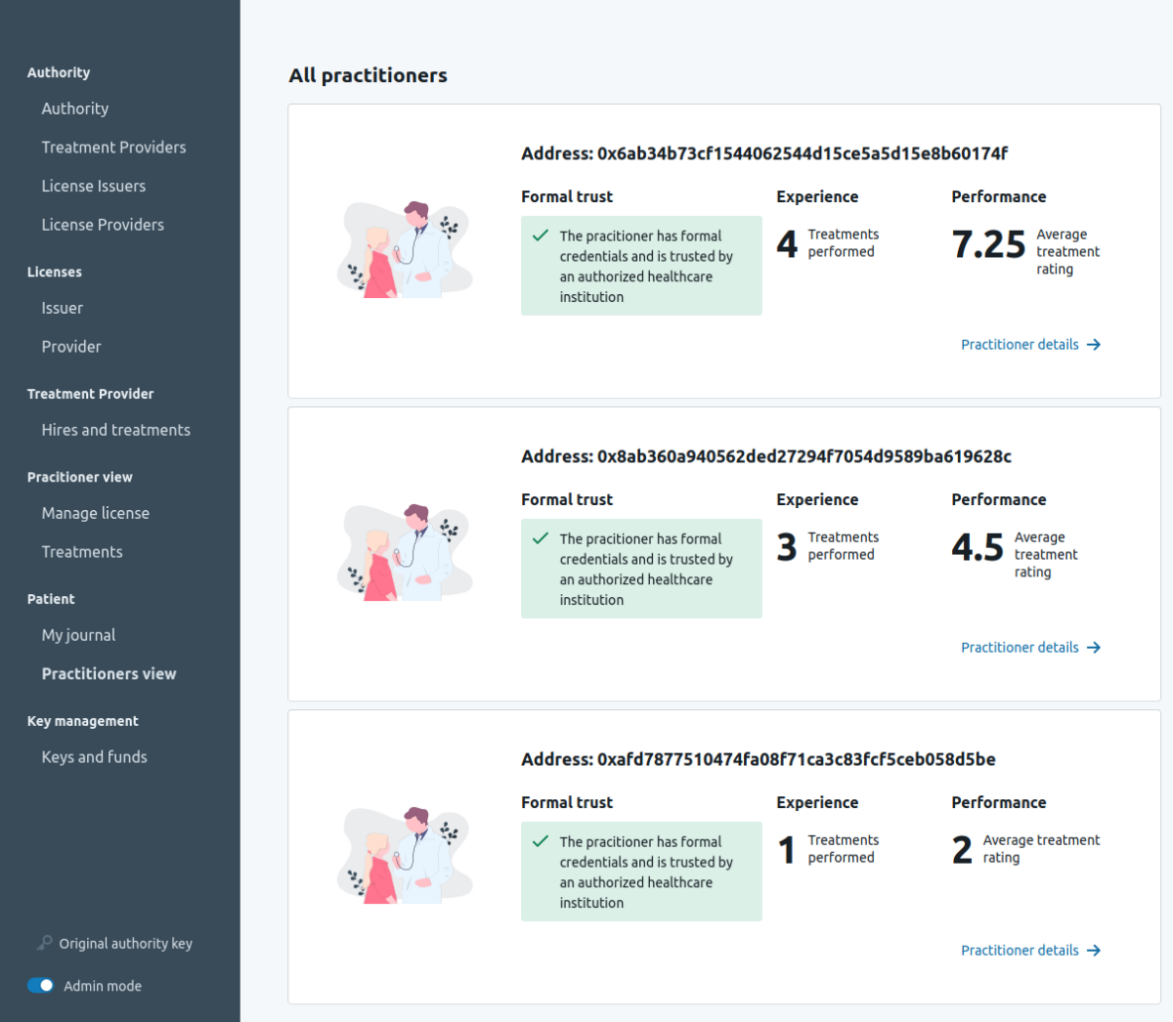
Verification overview of health practitioners.

Once a license is considered trusted through the relationships captured on the blockchain, patients can use this information to check it. When meeting a practitioner, they can use the procedures defined in the smart contracts to check if their license is trusted and valid. Figure 7 illustrates the verification of the license, experiences, and skills of health practitioners.

###### Evaluation of the Treatment

Once the treatment is completed, the patient may evaluate the treatment. The patient can do so via the one-time key generated during the treatment creation and thus create the evaluation without revealing their identity. This evaluation can be linked implicitly to a treatment provider and an approving practitioner. Future patients can use this information to enforce or decrease their trust in a practitioner.

###### Usage Outside of the Patient and Caregiver Relationship

**Figure 8:**
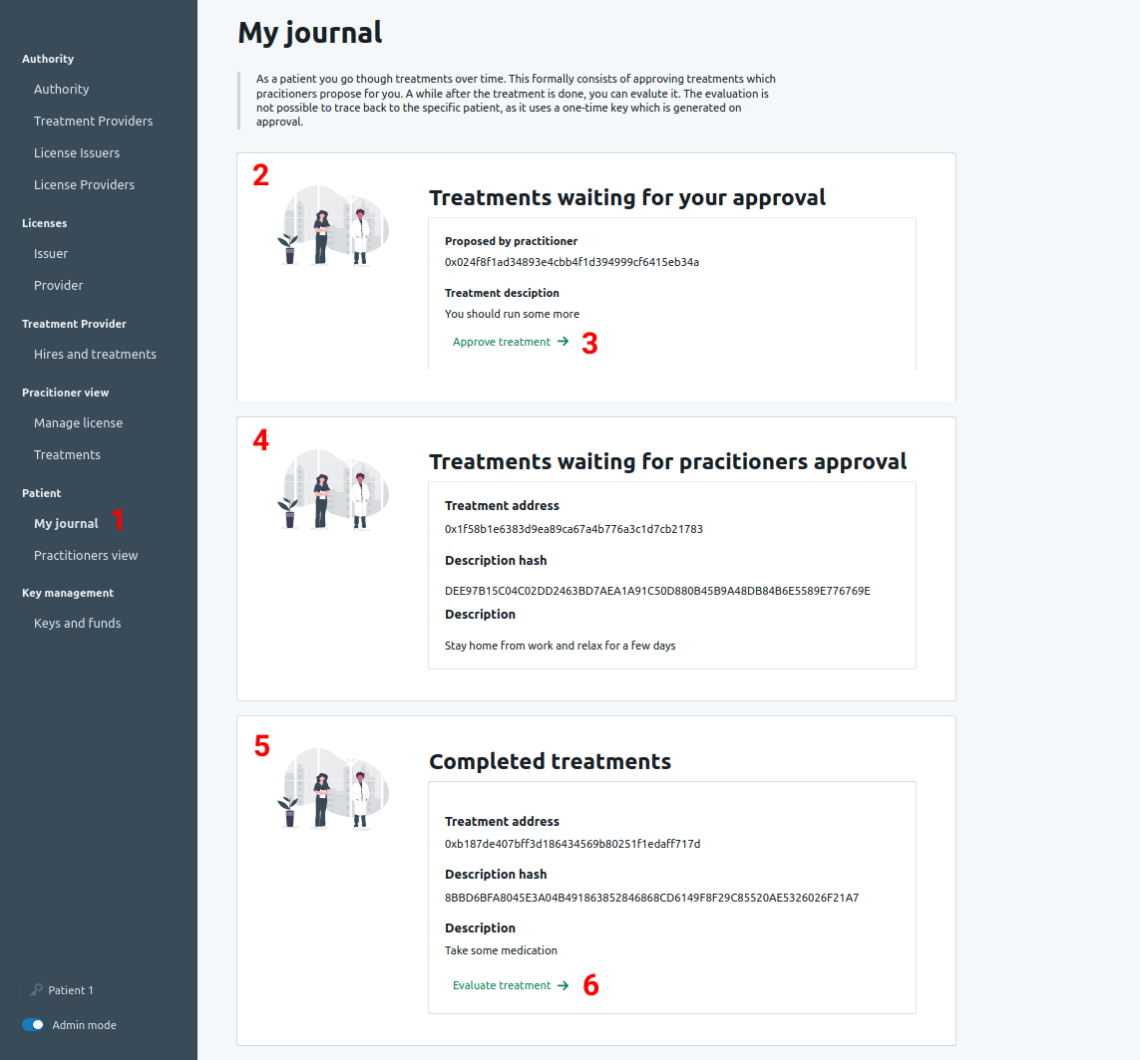
Overview of the user interface for patients.

Although we focus on the patient and caregiver relationship in the context of treatment, we also surface how public data sets have many use cases outside of this setting, such as audits, second opinion services, reporting, and evaluation of treatment providers. Figure 8 shows the user interface for the patients.

## Discussion

This work outlines a theoretical basis for the need for a blockchain enhanced trust model in a virtual health care setting, which contributes to the overall understanding of how the health care sector transforms into a new era, Healthcare 4.0, and the potential problems that could arise along with this transformation. Following this analysis, we built and implemented the novel VerifyMed platform that could trust in a virtualized health care environment.

We have used design principles from the Industry 4.0 concept to forecast Healthcare 4.0 and address an emerging problem in a future health care system. Our results show that our proof-of-concept implementation can be used to verify the authority of a health care worker, experience, and competence. The verifier does not have to place any trust in the health care workers themselves. This process can be performed by anyone with access to the Ethereum blockchain network, making the evaluation process fully transparent. In the further development of the system, microcredentialing can be incorporated, making it possible to verify specific skills among health care professionals.

Our trust model is justified in real-world governance of health care. As an environment with heavy regulatory oversight, capturing pre-existing governance relationships on a public blockchain serves as a natural first step for providing trust in virtualized settings. Furthermore, we strengthen our model by adding revocation abilities, where the trust of a governance entity can be revoked if it acts in bad faith. The result is a trust model justified in the inherited trust relationship between patients and the currently established health care system.

The VerifyMed platform enables individuals to store their respective credentials in a secure and accessible manner. The provenance of these data can be guaranteed using the immutability of the blockchain. In theory, this should mitigate the need to constantly verify the credentials from the issuing body and potentially speed up recruitment and onboarding processes in the health care sector.

We note that our trust model is extensible. A patient may trace all trust relationships from any evidence back to a top-level authority. The patient stands free to blindly trust the blockchain or use a third-party service to independently verify each of the upstream governance entities.

Health data are inherently sensitive, and thus, demand privacy. The management, storage, and access rights of health care data are highly regulated, both through general data protection acts such as the GDPR, health data specific acts such as the HIPAA, and often national health data laws. In an initial analysis [46], VerifyMed complies with the GDPR, although the general compliance of blockchain and the GDPR is under investigation; this work may have to be updated. Future work should include a comprehensive compliance analysis, and if appropriate, suggest an adaptation to comply with specific national health data laws and the HIPAA.

VerifyMed does not cover an identity solution for any of the users, and this is obviously an important component for the system to be ready for a real-life setting. As identity management is a core function in a health informatics system, future work must address this issue and develop an identity solution fitted to this particular use case.

VerifyMed uses the public Ethereum blockchain to host smart contracts. This choice is incorporated into the architecture, as the public nature of the blockchain is considered. Using a public blockchain requires limiting the published data to protect patient privacy, and access control schemes must be implemented within smart contracts. In addition, there is a need to incorporate a mechanism to transfer Ether (or smaller fractions of gas; ie, gwei or nanoeth) between accounts, thereby allowing them to submit transactions. The key advantage of using a public blockchain for this use case is transparency, no need for interorganizational agreements, and the possibility of interacting with the underlying cryptocurrency of Ethereum. The disadvantages of using the Ethereum blockchain are the monetary price of transactions and scalability issues related to low throughput. Furthermore, as the platform is governed by a set of authorities, license issuers, license providers, and treatment providers, this allows the publication of evidence for trust rooted in real-world trust relationships on the blockchain. This model contrasts with the fully trustless principles, which are usually applied within public blockchains but are necessary for the complex system of the health care domain. However, this can open up using a permissioned blockchain instead of fully public, which could have benefits such as reduced transaction costs and higher scalability. This should be explored in future studies.

VerifyMed could, with future updates, enrich the current trust model by including more trust requirements, such as (1) the caregiver must trust that the patient exists, (2) the caregiver must trust the authenticity of the data that the patient is willing to share, and (3) a third party (eg, an insurance company) must be able to trust the claim of the patient that care provision has taken place. The patient cannot really understand the credentials and experience of a caregiver because having a license is not the same as having credentials and having competency is not the same as having experience. Thus, the system should make the credentials or competency contextually important to the patient.

In the forecasting analysis, experts were consulted based on a convenience sample. This is not a comprehensive review of the general opinions of experts but just guidance in the direction of forecasting. It is not possible to preclude that this sample was not biased. However, a review of the literature supports input from expert consulting.

The trust mechanism that the blockchain enables in this concept provides a more transparent, accountable, and controlled handling of verifying competence and experience. This could also be achieved using a centralized solution. However, in the transition to Healthcare 4.0, decentralization is of increasing importance. This concept is consistent with this development.

Future research also needs to further validate the use case and the proof of concept of VerifyMed. Before modifying and updating the proof of concept, a feasibility study with real users should be undertaken to validate the concept and explore the interface design. The feasibility study could also address the challenge of how a patient interprets the presented verification of experience and verification of the competence of a caregiver.

## Abbreviations

dApps: decentralized applications
GDPR: General Data Protection Regulation
HIPAA: American Health Insurance Portability and Accountability Act
PROM: patient-reported outcome measure
